# Supplementary material for: Readiness of health posts to manage child wasting in six districts of Ethiopia
Source: PLoS One. 2025 Nov 20;20(11):e0320824. doi: 10.1371/journal.pone.0320824 (PMC12633898; doi:10.1371/journal.pone.0320824)
Supplement: Attachment — Submitted filename: Comments and Suggestions for Author.docx [file pone.0320824.s001.docx]

**Title:** **Readiness of Health Posts to Manage Wasting in Ethiopia.**

**Comments to Authors:**

The study looks interesting and original.

The use of a facility-based cross-sectional design is well-suited to assess service readiness.

The author should consider making the title more specific, action-oriented and specific with area and time.

**Abstract**

1. The methods section of the abstract is vague. There is insufficient detail on what specific domains or indicators were used in the "weighted additive approach" to assess readiness. A brief mention of assessment tools or core domains would improve scientific transparency.
2. The abstract should clarify why readiness is different for SAM vs. MAM. Without brief contextualization, the statistical difference lacks interpretive depth.
3. The conclusion introduces a strong caution against rapid integration of MAM services but does not adequately reflect the findings presented. Lacking evidence of poorly planned integration in the study design.

**Background/Introduction Section**

1. The authors effectively trace CMAM evolution and Ethiopia’s role, but references [1–8] are very old and back dated. Consider including more recent global reviews or evaluations post-2020 for contextual relevance.
2. Sentences like “Prioritizing children at the greatest risk of mortality…” (line 96) would benefit from direct attribution to a source or clearer explanation of mortality evidence.
3. The study objective is introduced at the very end (line 124). For clarity, move the aim earlier in the introduction and state it explicitly in one sentence.
4. Terms like “readiness,” “wasting,” and “integration” should be defined in operational terms as used in the study context.
5. While the study purpose is mentioned, a formally stated research question is missing. To what extent are health posts in pilot IMAM districts prepared to manage SAM and MAM?”
6. Abbreviations are needed to be placed in appropriate manner and introduced properly (e.g., SAM, MAM), though a full glossary.

**Methods:**

1. The study states that 6 IMAM districts were chosen “purposively” (line 157) but doesn’t explain the basis for selection. Were they representative? High/low burden? More detail is needed.
2. Within the districts, health posts were “randomly selected,” yet no method (e.g., lottery, systematic sampling) is described. Clarify the randomization process, as it contain discrepancies in sampling process
3. The regional diversity of the districts (Afar, Amhara, Oromia, etc.) is good for generalizability (line 141), but socio-demographic profiles or burden of malnutrition in these areas would strengthen the rationale.
4. The tools are only called “pre-tested” (line 162) with no detail on validation or structure. It’s important to specify what domains or questions were used and if they were aligned with WHO-SARA or other standards.
5. There is no clarification about the tool, its validity and measurements. Provide detail about the tool and how it was validated and was there any approving body for that tool?
6. The variables used in regression (line 190) are mentioned vaguely. More detail is needed: e.g., was distance continuous or categorical? Was “agrarian/pastoral” self-reported?
7. There's no mention of measures to minimize observer or selection bias (e.g., observer blinding, inter-rater reliability), or how missing data was handled.

**Results:**

1. The results provide clear comparative statistics between SAM and MAM readiness. Percentages and tracer items are well documented.
2. Figures are mentioned (Figure 1, 2), but not evaluated here. Assuming clarity, they complement the tabular results well.
3. Statements like “p < 0.001” (line 230) are given, but interpretation is minimal. Authors should explain implications—e.g., what does a statistically significant readiness gap mean in service delivery?
4. Results are presented as data points mostly already in table and figure form. There is need to provide the contextual information in the results section as well. For example, staff training is high (88.9%) but equipment availability is low (e.g., timers 16.7%). Such typer of arguments and contrasts are not highlighted.
5. The disaggregation by pastoral and agrarian areas is highly relevant, though details in results are thin. Include summary statistics (e.g., exact readiness percentages in pastoral areas).
6. The availability of commodities (67–69%) contrasts with basic amenities like electricity (40.3%). This should be flagged more clearly.
7. The table shows weighted scores but lacks 95% confidence intervals for domain-level results, which would improve interpretation of variability.

**Discussion:**

1. The discussion lacks comparative benchmarking. How do Ethiopia's results compare to readiness levels in similar LMIC contexts? This limits the paper’s global relevance.
2. The findings and arguments lack comparative benchmarking. How do Ethiopia's results compare to readiness levels in similar LMIC contexts? This limits the paper’s global relevance.
3. The discussion and analysis remains descriptive, not analytical. There is no in-depth discussion of *why* infrastructure gaps persist or which system bottlenecks are most critical. Use of a health systems framework (e.g., WHO building blocks) would have added rigor.
4. The authors acknowledge cross-sectional limitations but should also critique data reliability more strongly. The reliance on self-reported data with “limited verification” (line 354) is a major methodological weakness and reduces the study’s credibility.
5. The conclusion restates key findings on low readiness effectively. However, provide little or no offer **specific policy or programmatic recommendations** based on findings. For example, what type of integration approach or capacity-building measures would be feasible given current resource constraints?

**Comments to the Editor:**

This is a valuable contribution to understanding gaps in the decentralized health system's capacity to manage acute malnutrition in Ethiopia. However, the manuscript requires major revisions to improve its scientific rigor, particularly in its conceptual clarity, analytical depth, and linkage between data and interpretation.
