# Supplementary material for: Readiness of health posts to manage child wasting in six districts of Ethiopia
Source: PLoS One. 2025 Nov 20;20(11):e0320824. doi: 10.1371/journal.pone.0320824 (PMC12633898; doi:10.1371/journal.pone.0320824)
Supplement: Attachment — Submitted filename: PONE-D-25-08999.docx [file pone.0320824.s002.docx]

**Manuscript Number:** PONE-D-25-08999
**Article Type:** Research Article
**Full Title:** *Readiness of Health Posts to Manage Wasting in Ethiopia*

**Reviewer Comments-ZB**

**1. Title**

- The title requires clarification. Consider specifying that the focus is on *child* wasting, if applicable.
- Additionally, explain why the study is limited to *wasting* rather than addressing *child malnutrition* more broadly as mentioned in the introduction. This will help align reader expectations with the study scope.

**2. Abstract**

- The abstract lacks essential components, including a clear background, study objective, methodology, and key findings.
- Please revise it to include concise information on the study rationale, design, participant population, key results, and conclusions to make it informative and self-contained.

**3. Background**

- The background section does not adequately contextualize child nutrition interventions at the community level. It is important to describe the expected nutrition services or packages that health post staff are mandated to implement, as per national policy.
- A brief overview of Ethiopia’s community-level healthcare system would help readers unfamiliar with the setting understand the health post’s role.
- The concept of *readiness* is not clearly defined or conceptualized within the context of the study. Please provide a definition and explain its dimensions.
- The study objective is vague. Clarify what aspects of readiness are being assessed—e.g., infrastructure, human resources, supplies availability, etc.

**4. Methods**

- The study setting should be clearly described, including geographical, administrative, and health system context.
- The description of participants is insufficient. The sentence “This survey included health posts with health extension workers (n=72) in IMAM implementing areas” does not clarify who the actual respondents were. Were health extension workers surveyed? Were any supervisors or facility heads included?
- The sample size section is unclear. Please explain how the 72 health posts were selected, what sampling method was used, and how the sample size was determined.
- The readiness assessment tool—stated to be based on the Service Availability and Readiness Assessment (SARA)—needs more elaboration. How was it adapted? What domains or indicators were included? What scoring formats were used?
- The data collection process lacks critical details: Who were the data collectors? What qualifications did they have? Was any training or supervision provided?
- Operational definitions for key terms such as *wasting* and *readiness* must be clearly provided.
- If an index score was created, specify which domains were included and how they were weighted or combined. If Principal Component Analysis (PCA) was used, provide a detailed explanation of the technique and rationale.
- The parameters in the weighted formula for index creation must be clearly defined.
- The concept of "tracer items" is referenced in the analysis but not defined in the methods. This should be explained clearly.
- The phrase “The readiness percentage score for both datasets…” is confusing. What are these datasets? Clarification is needed.
- Overall, the analysis section lacks clarity and appears to proceed without explaining the basic assumptions and analytical framework.

**5. Conclusion**

- Due to significant methodological and measurement gaps, particularly in defining key concepts and clearly outlining the analytical approach, the credibility of the results and conclusions is currently compromised.
- Major revisions are needed to strengthen the study’s methodological rigor and transparency to support valid conclusions.
